# Psychosocial Consequences of Female Infertility in Iran: A Meta-Analysis

**DOI:** 10.3389/fpsyt.2020.518961

**Published:** 2020-11-05

**Authors:** Haniye Zarif Golbar Yazdi, Hamidreza Aghamohammadian Sharbaf, Hossein Kareshki, Malihe Amirian

**Affiliations:** ^1^Department of Psychology, Ferdowsi University of Mashhad, Mashhad, Iran; ^2^Department of Reproductive Medicine and Gynecology, Milad (Mashhad) Infertility Center, Mashhad, Iran

**Keywords:** infertility, psychosocial consequences, Iran, review, meta-analysis

## Abstract

**Background:** Although not a life-threatening condition, infertility does influence various aspects of life. Based on a meta-analysis of the relevant literature, the aim of this study is to identify the psychosocial consequences of infertility in Iranian women.

**Methods:** Comprehensive Portal of Human Sciences, Magiran, Scientific Information Database, Noormags, MEDLIB, ScienceDirect, Google Scholar, Medline, and ProQuest were the databases searched from inception (1999) to 2018. To maximize the comprehensiveness of the search, the reference lists of all the relevant papers identified were manually examined. The evaluation of the content was based on PRISMA guidelines, and Comprehensive Meta-Analysis software was used for data analysis.

**Results:** Based on the analysis of 124 quantitative papers, the psychosocial consequences of infertility in women in Iran can be classified into 14 categories: psychological well-being (effect size = 3.10), adaptation to infertility (effect size = 2.71), quality of life (effect size = 1.83), depression (effect size = 1.80), anxiety (effect size = 1.72), marital relationships (effect size = 1.37), personality disorders (effect size = 1.37), violence (effect size = 1.31), social support (effect size = 0.90), self-efficacy (effect size = 0.90), coping strategies (effect size = 0.84), irrational thoughts (effect size = 0.77), somatization disorders (effect size = 0.65), and sexual dysfunction (effect size = 0.55).

**Conclusion:** Considering the wide-ranging psychosocial consequences of infertility in women, it is necessary for treatment to account for psychological factors.

## Introduction

Infertility is characterized by the failure to achieve a clinical pregnancy despite 12 months of regular and unprotected sexual intercourse (1). The global prevalence of infertility is ~9–12.5% (2, 3). According to studies conducted in Iran, the total mean of infertility and the rate of primary infertility prevalence are 13.2% (4) and 17.3% (5), respectively; these figures are higher than the global average. Although not a life-threatening condition, infertility has intense psychosocial consequences. Infertility diagnosis and the subsequent treatment process usually impose excruciating stress on couples. Several studies have reported that couples with infertility are prone to experiencing depression (6), anxiety (7), sexual intercourse problems, marital problem (8, 9), decrease in self-confidence (10), and low levels of psychological well-being (11, 12) and quality of life (13).

Although infertility has an emotional impact on both partners, studies have shown that it imposes greater pressure on women, as demonstrated by the fact that 50% of infertile women considered this the biggest problem in their lives (14). In a previous study, many women with infertility stated that they could not imagine a life without children, while this was not the case with men (15). Further, it is mainly women who are subjected to fertility treatments, which serves to increase their psychological burden.

The stress and anxiety in women with infertility arises from issues such as missing out on the experience of motherhood, negative self-concept, and inability to continue the family line. Moreover, societal pressures are responsible for the extensive psychological consequences in women with infertility (16). In many cultures, infertility is perceived as something to be ashamed of (17). Specifically, owing to cultural and social factors as well as religious beliefs, having children is much more crucial in Asian compared to Western countries (18). In many traditional cultures, the male partners of women who are unable to bear children often remarry. In Iran, infertility can be considered a legal basis of divorce; that is, it is permitted upon the request of either partner (19).

Since infertility is an unexpected stress in the lives of couples, they are usually not equipped with the necessary information and appropriate coping strategies. Therefore, it is of paramount importance that psychological factors be taken into account during the treatment of infertility. Accordingly, the aim of this meta-analysis of studies concerning the psychological consequences of infertility in women is to provide experts with the data required to design therapy programs to preclude and decrease the negative effects of infertility in women. In this regard, this study seeks to answer the following question: What does the literature reveal about the psychological consequences of infertility in Iranian women?

## Materials and Methods

This study was conducted according to PRISMA guidelines. To investigate the entirety of the body of published research concerning the psychological consequences of infertility in women in Iran, the databases searched included Comprehensive Portal of Human Sciences, Magiran, Scientific Information Database, Noormags, MEDLIB, ScienceDirect, Google Scholar, Medline, and ProQuest. To maximize the comprehensiveness of the search, the reference lists for all the relevant papers were manually examined.

### Search Strategy

The search strategy was based on the PICOS model, as follows: P—infertile Iranian women, I—psychological interventions or assessment were done about psychosocial consequences of female's infertility, C—fertile Iranian people, O—psychosocial consequences of infertility, S—randomized control trial studies, pretest and posttest, quasi-experimental, and descriptive studies. To identify papers in the aforementioned electronic databases, the keywords used were “infertility,” “women's infertility,” “primary infertility,” “secondary infertility,” “quality of life,” “well-being,” “mood disorder,” “anxiety disorder,” “sexual dysfunction,” “psychological distress,” and combinations of these words.

### Inclusion and Exclusion Criteria

For studies to be included in this meta-analysis, they had to meet certain inclusion and exclusion criteria. The inclusion criteria were studies that (19) were published from inception (1999) to 2018, (20) investigated the psychological consequences of infertility, (21) reported sufficient data to measure effect sizes, (22) were in the form of full papers, published either online or accessible in library archives, and (23) had Iranian authors, although the text could be in either Persian or English. The exclusion criteria were studies that (19) did not provide full-text access, (20) did not report information necessary to measure effect sizes, and (21) were duplicate articles based on reviewing titles and abstracts.

### Statistical Analysis

In this study, we employed Hedges' effect size to quantitatively evaluate the results. To elaborate, we first drew a funnel plot to identify publication bias. Following the sensitivity analysis and exclusion of studies with publication bias, we determined the effect size of every psychosocial consequence of infertility in women in Iran as well as the effect size using fixed and random models. Further, to investigate the heterogeneity of the effect sizes in the initial studies, that is, those that did not make it to the final analysis stage despite originally meeting certain criteria, we employed Cochran's Q and the chi-l index. Comprehensive Meta-Analysis software (CMA; Biostat, Inc.) was used for data analysis.

## Results

**Figure 1:**
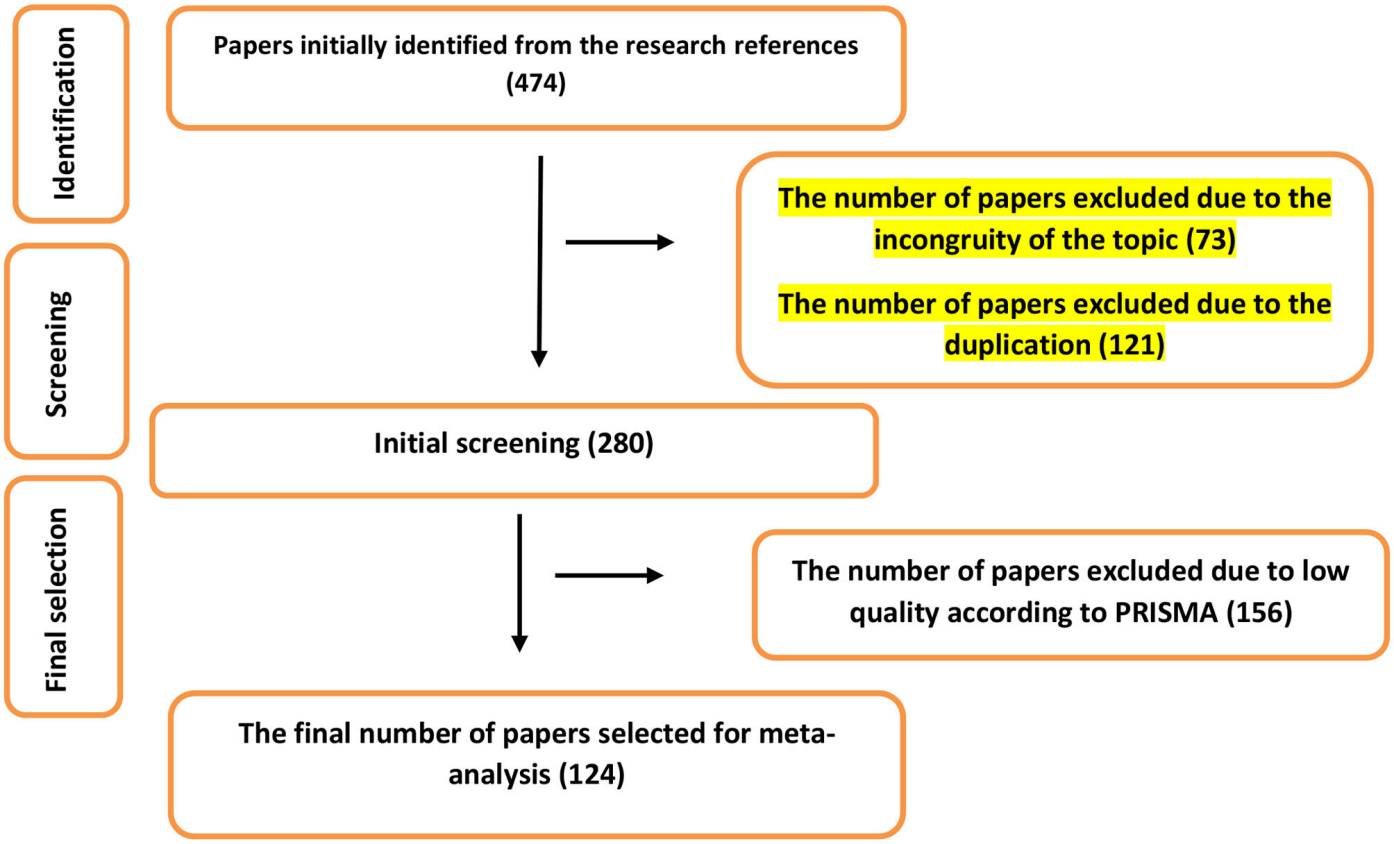
Flowchart of process of selecting papers for inclusion in the meta-analysis.

Out of the 474 papers initially identified in the electronic databases, 73 and 121 were excluded in the first stage owing to the incongruity of their topics and duplication. As a result, a total of 346 papers were selected for the next stage. In this stage, the authors investigated the texts of the papers. A total of 156 papers that had a low quality based on PRISMA principles were excluded from the list. At this point, 124 papers were selected for the meta-analytic process (Figure 1). Details on the studies are presented in Table 1.

**Table 1:** Summary of studies included in review.

**Psychosocial consequences**	**ID**	**References**	**Sample size**	**Age (Y)**	**Study type**	**Statistics**	***d***	**Sig**
Sexual dysfunction	1	(24)	120	24–38	Descriptive	T	0.27	0.00
	2	(25)	250	NA	Cross-sectional	T	2.16	0.00
	3	(26)	296	17–50	Cross-sectional	M-SD	0.44	0.00
	4	(27)	220	NA	Cross-sectional	M-SD	0.00	0.98
	5	(28)	32	NA	Field trial	M-SD	0.45	0.00
	6	(29)	100	NA	Descriptive	M-SD	0.52	0.01
	7	(30)	200	20–45	Descriptive	M-SD	0.16	0.40
	8	(31)	604	18–42	Cross-sectional	M-SD	0.45	0.00
	9	(32)	200	NA	Descriptive	T	0.98	0.00
						T	0.44	0.00
						T	0.53	0.00
						T	0.02	0.88
	10	(33)	200	22–45	Descriptive	T	0.08	0.23
	11	(34)	600	18–45	Descriptive	M-SD	0.10	0.37
	12	(35)	90	21–48	Descriptive	T	0.62	0.00
	13	(36)	600	NA	Descriptive	M-SD	0.06	0.59
	14	(37)	180	18–40	Descriptive	T	0.97	0.00
		**14 appropriate article for meta-analysis**					**M = 0.55**	
Depression	1	(11)	22	23–37	Semi-Experimental	M-*SD*	0.03	0.92
	2	(38)	200	NA	Descriptive	T	0.85	0.00
	3	(39)	30	20–40	Semi-Experimental	M-SD	0.20	0.53
	4	(40)	45	NA	Semi-Experimental	M-SD	1.89	0.00
						M-SD	0.52	0.00
	5	(41)	43	18–40	Descriptive	M-SD	1.74	0.00
	6	(42)	70	NA	Prospective	M-SD	0.13	0.41
	7	(43)	30	NA	Semi-Experimental	M-SD	1.57	0.00
	8	(44)	30	19–42	Semi-Experimental	M-SD	1.69	0.00
	9	(45)	30	NA	Semi-Experimental	M-SD	0.90	0.00
	10	(46)	80	NA	Descriptive	T	0.22	0.00
	11	(47)	40	NA	Semi-Experimental	T	1.23	0.00
	12	(48)	30	21–37	RTC	M-SD	0.56	0.13
						M-SD	0.51	0.16
	13	(49)	61	20–40	RTC	T	3.97	0.00
	14	(50)	23-42	70	Descriptive	M-SD	0.50	0.00
	15	(51)	90	NA	Descriptive	M-SD	0.90	0.00
	16	(52)	40	NA	Semi-Experimental	M-SD	3.62	0.00
						M-SD	3.15	0.00
	17	(53)	294	NA	Descriptive	M-SD	0.41	0.00
	18	(54)	31	20–35	RTC	M-SD	1.45	0.00
	19	(55)	40	24–40	Semi-Experimental	M-SD	3.53	0.00
						M-SD	5.75	0.00
	20	(56)	40	NA	Semi-Experimental	M-SD	2.05	0.00
						M-SD	2.42	0.00
	21	(57)	89	NA	RTC	M-SD	1.75	0.00
	22	(36)	600	NA	Descriptive	M-SD	0.42	0.00
	23	(58)	174	NA	Descriptive	T	0.73	0.00
	24	(59)	300	17–45	Descriptive	M-SD	0.60	0.00
		**24 appropriate article for meta-analysis**					**M = 1.80**	
Marital satisfaction	1	(60)	282	NA	Descriptive	R	0.40	0.00
						R	0.87	0.00
						R	0.62	0.00
	2	(61)	90	25–44	Descriptive	R	1.85	0.00
	3	(62)	24	NA	Semi-Experimental	M-SD	1.84	0.00
	4	(63)	40	NA	Semi-Experimental	M-SD	3.29	0.00
	5	(64)	139	20–50	Descriptive	M-SD	1.54	0.00
	6	(65)	32	25–40	Semi-Experimental	M-SD	1.32	0.00
	7	(66)	36	NA	RTC	T	0.70	0.05
	8	(67)	40	22–47	Semi-Experimental	M-SD	2.35	0.00
						M-SD	1.88	0.00
	9	(68)	30	NA	Semi-Experimental	M-SD	1.21	0.00
	10	(69)	72	NA	RTC	M-SD	1.94	0.00
						M-SD	2.39	0.00
	11	(70)	64	NA	Semi-Experimental	M-SD	0.93	0.00
						M-SD	1.73	0.00
	12	(71)	20	NA	Semi-Experimental	M-SD	0.34	0.43
	13	(72)	220	NA	Descriptive	T	0.47	0.00
						T	0.61	0.00
	14	(73)	100	18-43	Descriptive	R	0.79	0.00
						R	0.22	0.1.
	15	(74)	100	NA	Descriptive	T	0.92	0.00
						T	0.90	0.00
	16	(75) ^•^	40	NA	Semi-Experimental	M-SD	14.0	0.00
	17	(76)	220	NA	Descriptive	M-SD	0.06	0.64
	18	(22)	520	NA	Descriptive	M-SD	0.22	0.07
						M-SD	0.23	0.05
						M-SD	0.39	0.00
	19	(46)	80	NA	Descriptive	T	0.08	0.54
	20	(77)	30	NA	Semi-Experimental	M-SD	0.63	0.08
	21	(78)	24	NA	Semi-Experimental	M-SD	3.48	0.00
	22	(79)	186	NA	Descriptive	M-SD	1.10	0.00
	23	(80)	80	NA	Descriptive	M-SD	0.27	0.01
	24	(78)	130	18–37	Descriptive	T	0.36	0.04
	25	(81)	130	20–40	Descriptive	R	0.74	0.00
	26	(82)	100	NA	RTC	M-SD	0.49	0.00
	27	(83)	24	25–35	Semi-Experimental	M-SD	0.73	0.08
						M-SD	0.83	0.04
	28	(84)	440	NA	Descriptive	T	0.35	0.01
	29	(85)	60	NA	Semi-Experimental	M-SD	0.71	0.05
	30	(86)	198	NA	Descriptive	M-SD	0.14	0.50
	**30 appropriate article for meta-analysis**						**M = 1.37**	
Anxiety	1	(40)	45	NA	Semi-Experimental	M-SD	3.77	0.00
	2	(87)	30	NA	Semi-Experimental	M-SD	1.62	0.00
	3	(20)	30	25–40	Semi-Experimental	M-SD	1.39	0.00
	4	(41)	43	18–40	Descriptive	M-SD	2.04	0.00
	5	(64)	139	20–50	Descriptive	M-SD	1.54	0.00
	6	(88)	30	20–35	Descriptive	T	0.80	0.00
	7	(89)	108	18–40	RTC	M-SD	0.57	0.00
						M-SD	0.47	0.01
	8	(43)	30	NA	Semi-Experimental	M-SD	1.92	0.00
						M-SD	1.99	0.00
	9	(42)	70	NA	Prospective	M-SD	0.02	0.97
	10	(90)	50	20–45	RTC	M-SD	0.97	0.00
	11	(91)	100	25–35	RTC	T	0.26	0.18
	12	(46)	80	NA	Descriptive	T	0.20	0.01
	13	(49)	61	20–40	RTC	T	2.37	0.00
						T	2.76	0.00
	14	(50)	23–42	70	Descriptive	M-SD	0.95	0.00
	15	(92)	65	20–49	Semi-Experimental	T	0.37	0.00
	16	(54)	31	20–35	RTC	M-SD	2.37	0.00
						M-SD	2.76	0.00
	17	(93)	24	NA	RTC	M-SD	2.04	0.00
	18	(94)	22	22–37	Semi-Experimental	M-SD	2.69	0.00
	19	(95)	130	18–37	Descriptive	T	0.83	0.00
	20	(55)	40	24–40	Semi-Experimental	M-SD	1.48	0.00
						M-SD	2.72	0.00
	21	(96)	60	20–45	RTC	T	0.59	0.02
	22	(97)	76	18–35	Semi-Experimental	T	0.09	0.70
	23	(98)	30	20–40	RTC	M-SD	2.92	0.00
	24	(57)	89	NA	RTC	M-SD	0.72	0.00
						M-SD	1.93	0.00
	25	(59)	300	17–45	Descriptive	M-SD	0.13	0.23
	26	(99)	60	NA	Semi-Experimental	T	0.84	0.00
	27	(100)	80	20–44	Descriptive	T	1.52	0.00
	28	(101)	110	20–40	RTC	M-SD	1.31	0.00
	**28 appropriate article for meta-analysis**						**M = 1.72**	
Physical complaints	1	(41)	43	18–40	Descriptive	M-SD	0.49	0.02
	2	(42)	70	NA	Prospective	M-SD	0.00	0.01
	3	(50)	23-42	70	Descriptive	M-SD	3.05	0.00
	4	(95)	130	18–37	Descriptive	T	0.34	0.05
	5	(18)	150	17–45	Descriptive	M-SD	0.00	0.94
	6	(102)	240	NA	Descriptive	M-SD	0.95	0.00
	7	(59)	300	17–45	Descriptive	M-SD	0.01	0.89
	8	(103)	100	25–45	Descriptive	T	0.39	0.00
	**8 appropriate article for meta-analysis**						**M = 0.65**	
Social support	1	(62)	24	NA	Semi-Experimental	M-SD	1.68	0.00
	2	(65) ^•^	32	25–40	Semi-Experimental	M-SD	8.08	0.00
	3	(42)	70	NA	Prospective	M-SD	0.02	0.92
	4	(104)	90	NA	Descriptive	M-SD	0.55	0.03
	5	(105)	80	24–45	Semi-Experimental	T	0.51	0.02
	6	(106)	40	NA	Descriptive	T	0.56	0.07
	7	(72)	220	NA	Descriptive	T	0.04	0.72
	8	(107)	200	19–59	Descriptive	R	0.98	0.00
	9	(77)	30	NA	Semi-Experimental	M-SD	0.63	0.08
	10	(50)	23-42	70	Descriptive	M-SD	3.05	0.00
	11	(95)	130	18–37	Descriptive	T	0.30	0.08
	12	(53)	294	NA	Descriptive	M-SD	0.85	0.00
	13	(108)	280	NA	Descriptive	M-SD	0.98	0.00
	14	(109)	150	NA	Descriptive	R	0.02	0.90
	15	(18)	150	17–45	Descriptive	M-SD	3.05	0.00
	16	(102)	240	NA	Descriptive	M-SD	1.07	0.00
	17	(85)	60	NA	Semi-Experimental	M-SD	0.35	0.35
	18	(103)	100	25–45	Descriptive	T	0.77	0.00
		**18 appropriate article for meta-analysis**					**M = 0.90**	
Coping strategies	1	(110)	400	NA	Descriptive	M-SD	0.81	0.00
	2	(111)	266		Descriptive	T	0.10	0.43
	3	(112)	160	20–40	Descriptive	M-SD	0.37	0.01
	4	(105)	80	24–45	Semi-Experimental	T	0.18	0.41
	5	(75)	40	NA	Semi-Experimental	M-SD	2.81	0.00
	6	(113)	40	NA	Semi-Experimental	M-SD	1.26	0.00
	7	(114)	200	NA	Descriptive	M-SD	0.30	0.13
						M-SD	0.10	0.60
		**7 appropriate article for meta-analysis**					**M = 0.84**	
Adjustment	1	(68)	30	NA	Semi-Experimental	M-SD	1.21	0.00
	2	(70)	64	NA	Semi-Experimental	M-SD	1.35	0.00
						M-SD	2.07	0.00
	3	(115)	92	20–35	RTC	M-SD	2.73	0.00
	4	(78)	24	NA	Semi-Experimental	M-SD	3.48	0.00
		**4 appropriate article for meta-analysis**					**M = 2.71**	
Violence	1	(38)	200	NA	Descriptive	T	2.27	0.00
	2	(116)	32	NA	Semi-Experimental	M-SD	2.74	0.00
	3	(32)	200	NA	Descriptive	T	0.98	0.00
						T	0.43	0.00
						T	0.53	0.00
						T	0.02	0.98
	4	(117)	200	NA	Descriptive	T	0.86	0.00
						T	0.48	0.00
						T	0.53	0.00
						T	0.01	0.92
	5	(18)	150	17–45	Descriptive	M-SD	0.15	0.19
	6	(102)	240	NA	Descriptive	M-SD	0.96	0.00
	7	(59)	300	17–45	Descriptive	M-SD	0.16	0.14
	8	(103)	100	25–45	Descriptive	T	0.41	0.00
		**8 appropriate article for meta-analysis**					**M = 1.31**	
Quality of life	1	(118)	60	20–40	RTC	M-SD	2.51	0.00
	2	(119)	11	18–23	Semi-Experimental	M-SD	1.83	0.00
	3	(66)	36	NA	RTC	T	0.69	0.05
	4	(120)	200	15–49	Descriptive	T	0.45	0.00
	5	(121)	190	20–45	Descriptive	R	1.55	0.00
	6	(122)	450	15–49	Descriptive	M-SD	0.32	0.00
	7	(21)	276	NA	Descriptive	T	0.04	0.69
	8	(123)	190	20–45	Descriptive	R	0.5	0.00
	9	(124)	29	NA	RTC	M-SD	1.71	0.00
	10	(125)	45	NA	RTC	M-SD	1.59	0.00
						M-SD	1.48	0.00
	11	(51)	90	NA	Descriptive	M-SD	1.91	0.00
	12	(79)	186	NA	Descriptive	M-SD	0.30	0.00
	13	(126)	40	25–45	Semi-Experimental	M-SD	0.40	0.20
						M-SD	2.84	0.00
						M-SD	0.92	0.00
						M-SD	0.70	0.03
						M-SD	1.19	0.00
						M-SD	1.14	0.00
						M-SD	1.44	0.00
	14	(33)	200	22–45	Descriptive	M-SD	0.03	0.79
	15	(127)	24	25–35	Semi-Experimental	M-SD	2.11	0.00
						M-SD	2.65	0.00
	16	(23)	120	NA	Descriptive	M-SD	2.14	0.00
	17	(128)	79	20–40	Descriptive	M-SD	0.64	0.00
		**17 appropriate article for meta-analysis**					**M = 1.83**	
Irrational beliefs	1	(22)	260	18–45	Descriptive	M-SD	0.24	0.05
	2	(93)	24	NA	RTC	M-SD	1.77	0.00
	3	(129)	100	NA	Descriptive	M-SD	0.16	0.09
	4	(108)	280	NA	Descriptive	M-SD	0.98	0.00
	5	(85)	60	NA	Semi-Experimental	M-SD	0.71	0.05
		**5 appropriate article for meta-analysis**					**M = 0.77**	
Self-efficacy	1	(130)	53	NA	Descriptive	M-SD	0.12	0.53
	2	(131)	104	20–45	RTC	T	2.11	0.00
	3	(132)	200		Descriptive	M-SD	0.49	0.00
		**3 appropriate article for meta-analysis**					**M = 0.90**	
Personality disorders	1	(133)	92	NA	Descriptive	T	0.10	0.59
	2	(59)	300	17–45	Descriptive	M-SD	0.28	0.01
	3	(134)	14	NA	Semi-Experimental	T	3.73	0.00
		**3 appropriate article for meta-analysis**					**M = 1.37**	
Well-being	1	(135)	45	NA	Semi-Experimental	M-SD	1.49	0.00
						M-SD	1.54	0.00
	2	(63)	40	NA	Semi-Experimental	M-SD	1.50	0.00
	3	(71)	20	NA	Semi-Experimental	M-SD	4.06	0.00
						M-SD	4.87	0.00
	4	(104)	90	NA	Descriptive	M-SD	1.25	0.00
	5	(136)	197	NA	Descriptive	M-SD	3.58	0.00
	6	(137)	30	NA	Semi-Experimental	T	1.13	0.00
	7	(138) ^•^	16	NA	Semi-Experimental	M-SD	6.88	0.00
	8	(139)	22	NA	Semi-Experimental	M-SD	1.51	0.00
	9	(75)	40	NA	Semi-Experimental	M-SD	2.82	0.00
	10	(125)	45	NA	RTC	M-SD	2.35	0.00
						M-SD	1.82	0.00
	11	(140)	24	NA	RTC	M-SD	2.03	0.00
	12	(80)	80	NA	Descriptive	M-SD	0.77	0.01
	13	(94)	22	22–37	Semi-Experimental	M-SD	3.04	0.00
	14	(54)	31	20–35	RTC	M-SD	2.06	0.00
	15	(141)	24		Semi-Experimental	M-SD	3.21	0.00
						M-SD	3.83	0.00
		**15 appropriate article for meta-analysis**						***M*** **= 3.10**	

• *The research, which had distribution bias, was subsequently discarded*.

A total of 292 effect sizes from the studies that initially entered the meta-analysis were measured. The reason for the number of effect sizes being greater than the number of included studies was the fact that every study contained numerous variables related to the psychosocial consequences of infertility. Since one of the main assumptions of our meta-analysis was the absence of publication bias, we first employed a graphic method (funnel plot) to identify publication bias and eliminate those studies.

**Figure 2:**
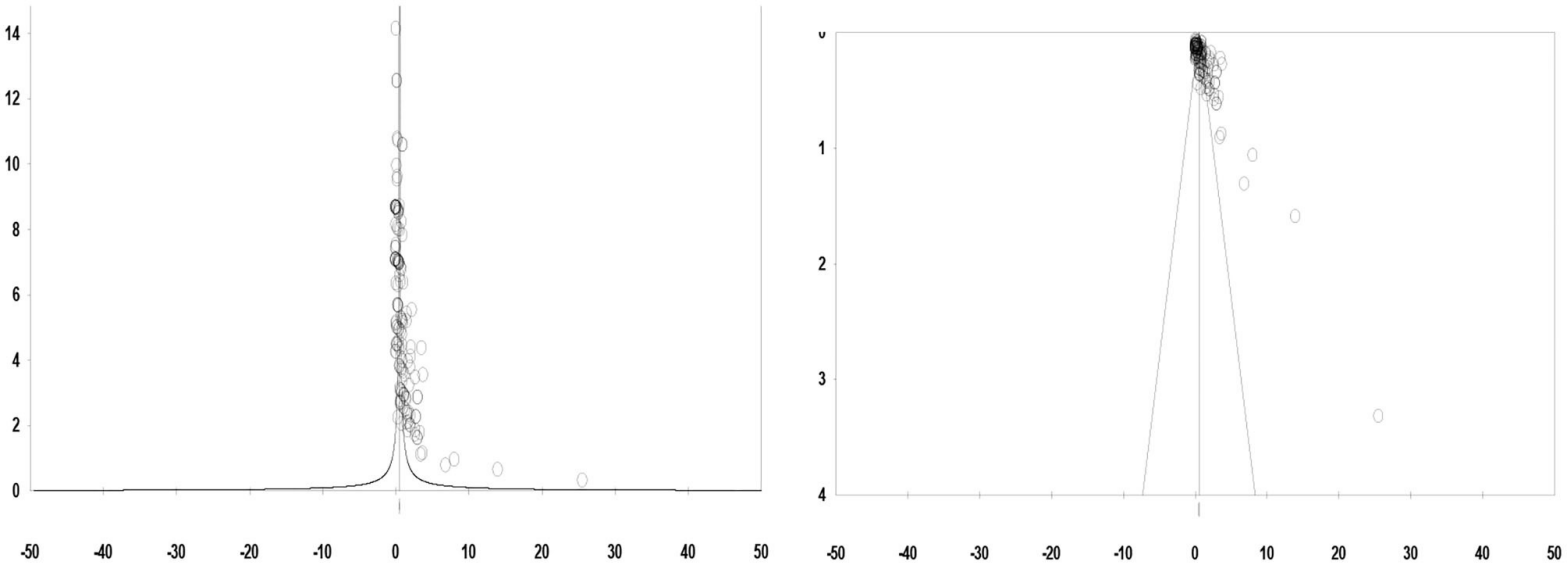
Funnel plot for publication bias before **(Left)** and after **(Right)** sensitivity analysis.

By observing the funnel plot (Figure 2), it can be seen that the points are not distributed symmetrically around the plot, owing to the uncommon and deviated values of the effect sizes. Further elimination of three effect sizes led to a symmetrical shape in the funnel plot (Figure 2). Finally, from the 124 papers determined to be appropriate for meta-analysis, a total of 243 effect sizes regarding the psychosocial consequences of infertility in women were identified.

Table 2 presents the number of papers for each psychosocial consequence, and their mean effect sizes, according to Hedges' effect size index. Based on the results presented in Table 1, it is evident that the maximum effect size−3.10—relates to psychological well-being. Therefore, it can be deduced that the most significant psychosocial consequence of infertility among women is the psychological well-being variable and its related factors. In addition, the minimum Hedges' effect size−0.55—relates to sexual dysfunction. Thus, sexual dysfunction is the least significant psychosocial consequence of infertility in women.

**Table 2:** Effect sizes of psychosocial consequences of infertility in women in Iran.

**Psychosocial outcomes**	**Hedges' g**	**Hedges' g and 95% CI**										
		**−3.00**	**−2.00**	**1.00−**	**0.00**		**1.00**		**2.00**		**3.00**	
Sexual dysfunction	0.55				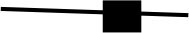						
Depression	1.80				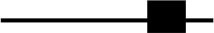							
Marital satisfaction	1.37				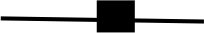							
Anxiety	1.72				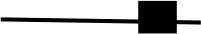							
Physical complaints	0.65		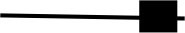									
Social support	0.90		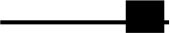									
Coping strategies	0.84		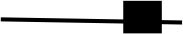									
Adjustment	2.71							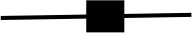			
Violence	1.31					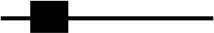					
Quality of life	1.83					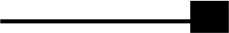					
Irrational beliefs	0.77	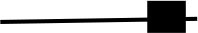									
Self-efficacy	0.90			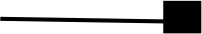								
Personality disorders	1.37					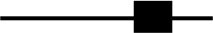					
Well-being	3.10									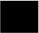	
Fixed overall	0.50			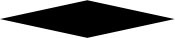							
Random overall	0.83			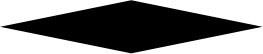							

*CI, confidence interval*.

Table 3 illustrates the combined effect of the fixed and random models in Iranian studies on the psychosocial consequences of female infertility after sensitivity analysis. The means of the combined effect sizes for the psychosocial consequences of female infertility in fixed and random models were 0.58 and 1.03, respectively, both of which were statistically significant (*P* ≤ 0.00).

**Table 3:** Hedges' effect sizes of fixed and random models related to psychosocial consequences of infertility.

**Model**		**Number of effect sizes**	**Hedges' effect sizes (g)**	**Standard error**	**95% CI**		***Z*-value**	***P***
					**Lower limit**	**Upper limit**		
After sensitivity analysis	Fixed	243	0.58	0.01	0.55	0.60	48.41	0.00
	Random	243	1.03	0.04	0.95	1.12	23.81	0.00

To specify the final meta-analysis model, a set of heterogeneity tests had to be conducted to ensure the presence of moderating variables. To examine the heterogeneity of the effect sizes in the studies, the Egger's test, Cochran's Q and Chi-I indices were employed. Egger's regression intercept tests revealed no evidence of publication bias (ρ = 0.24). The value obtained for the Q index (for 243 effect sizes) was 2,777.90, with a 242 degree of freedom, which was statistically significant (*P* ≤ 0.00) and indicated an actual difference between the effect sizes of the initial studies. Furthermore, the Chi-I results indicated that 91.28% of the variance present in the initial study results was real and could be obtained using moderating variables. Based on the criteria laid down by Bornstein, Hedges, Higgins, and Rothstein (142), a high heterogeneity is indicated in the initial studies. Based on the two heterogeneity indices, it was determined that the moderating variables played significant roles in the importance of the psychosocial consequences in women with infertility. Therefore, the random model was selected for meta-analysis, 1.03 was considered to be the combined effect size.

## Discussion

In this study, we performed a meta-analysis of Iranian studies on the psychological consequences of infertility in women. The aim was to synthesize the findings available in the literature, along with facilitating more precise future conclusions and presenting the possibility of devising plans to preclude, or at least decrease, such consequences.

As the results indicate, well-being is the psychological aspect most significantly impacted by infertility in women, with 19 significant effect sizes and a Hedges' effect size of 3.10. This is noteworthy, since existing studies have placed more emphasis on negative emotional factors than positive emotional factors. According to the definition provided by the World Health Organization, health is a state characterized by more than just the absence of disease. Therefore, a comprehensive definition of health should also include positive criteria such as welfare and physical, psychological, and social well-being (143). Infertility can decrease well-being by introducing stress in the personal, social, and marital domains. Indeed, when faced with the possibility of infertility, the situation is one of ambiguity: while the desire to bear a child lingers, its fulfillment is unlikely. While this is enough to impose great stress on couples, the side effects of assisted reproductive technology and the possibility of the treatment failing lower the well-being of women with infertility even further.

Adaptation to infertility emerged as the second most significant psychological consequence of infertility in women, with 12 significant effect sizes and a Hedges' effect size of 2.71. Adaptation to infertility refers to the cognitive and behavioral solutions individuals with infertility employ to cope with the crisis (144). The level of adaptation in couples with infertility is influenced by social and personal factors. Research has revealed that factors such as couples' attachment level, relationship quality, personal beliefs, and social support can influence adaptation to infertility (107). Owing to cultural and social reasons as well as religious beliefs, having children is very important for women in Islamic countries, and families generally expect married women to conceive within the first few months of marriage. As previously mentioned, in many traditional cultures, there is a high possibility of the male partners of women with infertility getting remarried (145). This can impose more psychological stress on women, decreasing their adaptation to infertility.

The third psychological aspect affected by infertility is quality of life, with 25 significant effect sizes and a Hedges' effect size of 1.83. In general, infertility and its treatment have negative effects on the quality of life. The severity of these effects is such that researchers have assigned it a distinct definition: fertility quality of life (146). In the context of 21st-century diseases that have a negative effect on quality of life, infertility is ranked third after cancer and cardiovascular diseases (147). Quality of life in women with infertility is related to factors such as economic status, income, and residential region (urban or rural) (148).

The results indicate that depression is the fourth psychological consequence of infertility in women, with 29 significant effect sizes and a Hedges' effect size of 1.80. At ~30.5%, the prevalence of depression in couples with infertility is higher than in the general public. Depression in individuals with infertility is related to a number of factors including gender (being female), duration of infertility, success/failure of previous treatments, and the cause of infertility (i.e., which of the partners is experiencing infertility) (149). In this regard, it is noteworthy that in the intervals between treatments, monthly variations in hopefulness or disappointment induce extensive psychological pressure in individuals with infertility. This pressure is more complex in women compared to men, mainly because women have a yearning for motherhood because of its link to their identity and meaning of life and are generally capable of making great sacrifices for the sake of childbearing. Aside from these psychological factors, having children is considered a source of power in women, in the context of not only the family but also of society. Therefore, when infertility deprives women of this source of power, it is natural that they experience pain and face problems at the familial and societal levels.

The fifth psychological consequence of infertility in women is anxiety, with 33 significant effect sizes and a Hedges' effect size of 1.72. The psychological, social, and financial challenges of infertility and its treatment can intensely influence the lives of couples. In the ranking of the worst possible events in a woman's life, infertility was positioned fourth, following death of parents and betrayal by the partner (150). A study in Iran revealed that the prevalence of anxiety in individuals with infertility is 33%, which is significantly higher than in the general population. Further, women have been reported to be 2.26 times more likely than men to report symptoms of anxiety (151).

Marital satisfaction is the sixth psychological consequence of infertility in women, with 41 significant effect sizes and a Hedges' effect size of 1.37. The stress of infertility affects marital adaptation, marital quality, and marriage stability. Marital quality and satisfaction in couples with infertility is significant, since it facilitates the continuation of infertility treatments and increases their chances of success. It is worth mentioning that the quality of a marital relationship is a major predictor of psychological health in women with infertility, and plays a crucial role in reducing their anxiety and depression levels (152).

The seventh psychological consequence of infertility in women relates to personality disorders, with seven significant effect sizes and a Hedges' effect size of 1.37. An individual's personality is influenced by infertility and its consequences, because people face problems differently and according to their personality traits. One of the factors that influence infertility and its consequences is the personality of individuals, because people with different personality traits face problem differently. Several studies have reported a high prevalence of personality disorders in women with infertility (59).

The eighth psychological consequence of infertility is violence, with 14 significant effect sizes and a Hedges' effect size of 1.31. According to the United Nations' Declaration on the Elimination of Violence Against Women in 1993, violence against women is defined as “any act of gender-based violence that results in physical, sexual, or mental harm or suffering to women, including threats of such acts, coercion, or arbitrary deprivation of liberty, whether occurring in public or private life” (153). Infertility is the main reason for violence against women. In a study conducted on 400 women with infertility in Iran, 61.8% had experienced domestic violence owing to infertility (145). Moreover, violence against women with infertility is associated with their partner's unemployment and insufficient education, as well as the forced nature of the marriage (154).

Social support is the ninth most influential psychocognitive consequence of infertility in women, with 18 significant effect sizes and a Hedges' effect size of 0.90. Social support is defined as a person's receipt of information, financial aid, health recommendations, and emotional support from individuals in their social network, including partners, relatives, and friends. The results of a qualitative study indicated that the four most necessary support categories in couples with infertility are social, financial, spiritual, and informational (155).

Self-efficacy ranks 10th in the list of psychological consequences of infertility in women, with five significant effect sizes and a Hedges' effect size of 0.90. Self-efficacy stems from the difference between how an individual perceives him/herself (self-concept) and the ideal-self. A small difference between these two leads to high self-efficacy, while larger differences result in low self-efficacy (156). Studies have shown that failure in performing “duties,” such as reproduction and fertility, decreases self-confidence. Therefore, having low self-efficacy decreases the level of psychological health and self-efficacy in women with infertility (157).

The use of inefficient coping strategies ranks 11th among the psychocognitive consequences of infertility in women, with eight significant effect sizes and a Hedges' effect size of 0.84. Coping strategies refer to mindful behaviors and cognitive attempts to manage current or expected stressors and negative events. These strategies are often categorized into two main groups, namely, problem-focused and emotion-focused. While problem-focused strategies employ behaviors such as acting and planning, emotion-focused strategies include expressing emotions and changing expectations (158). The results of Jafarzadeh et al.'s (159) study demonstrated that the cause of infertility is the main factor women without children consider while choosing the strategy to employ. For instance, when the male partner is infertile, women employ problem-focused coping strategies, whereas when they themselves experience infertility, they generally employ emotion-focused coping strategies (159).

Irrational thoughts regarding either having or not having children are the 12th most important psychological consequence of infertility, with five significant effect sizes and a Hedges' effect size of 0.77. Irrational thoughts are the beliefs that individuals learn in life. However, they are not fundamentally realistic and usual. Studies have shown that irrational cognitions regarding childbearing being the essence of a happy life are the main predictors of quality of life in couples with infertility (22).

Physical complaints are the 13th most significant psychocognitive consequence of infertility in women, with 10 significant effect sizes and a Hedges' effect size of 0.65. Overall, women with infertility have poorer psychological health compared to those who can bear children. A study showed that among the subscales of the Symptom Checklist-90-Revised, the highest mean score among women with infertility was for somatization (50). Stressful events can have a prominent role in the somatization aspect (160). Infertility, reported to be the most challenging event in a woman's life, results in a variety of physical complaints.

Finally, sexual dysfunction is the 14th psychological consequence of infertility, with 17 significant effect sizes and a Hedges' effect size of 0.55. Negative psychological and physiological consequences of the diagnosis and treatment of infertility can influence sexual functioning and lead to sexual dysfunction. Pregnancy, in the minds of many individuals, is the result solely of sexual intercourse and an indication of intimacy. Therefore, when pregnancy does not occur, it can discourage individuals from having sexual intercourse. On the contrary, in the process of infertility treatment, physicians generally prescribe a certain schedule for couples to have sexual intercourse. When physicians recommend a strict regimen regarding the timing of sexual intercourse and dictate details, it changes from an enjoyable act to a mechanical one (161). As reported, the prevalence of sexual dysfunction in couples with infertility is as high as 87.1% (162).

The act of fertilization and delivering a healthy child is considered the main event in the life of every couple. However, there is a significant factor that obstructs such joy: infertility. Although both men and women can experience infertility, social (and occasionally religious) pressures imposed on women often result in them carrying the burden of infertility, in turn experiencing psychological and physical problems. Further, therapeutic protocols must be adjusted to decrease, if not eliminate, these consequences.

## Data Availability Statement

All datasets generated for this study are included in the article/supplementary material.

## Author Contributions

HZ, HA, and HK contributed conception and design of the study. HZ and HA organized the database. HZ and HK performed the statistical analysis. HZ wrote the first draft of the manuscript. HZ and HA wrote sections of the manuscript. All authors contributed to manuscript revision, read, and approved the submitted version.

## Conflict of Interest

The authors declare that the research was conducted in the absence of any commercial or financial relationships that could be construed as a potential conflict of interest.
